# New Appliances and Things Medical

**Published:** 1895-11-23

**Authors:** 


### NEW APPLIANCES AND THINGS MEDICAL.

[We shall be glad to receive, at our Office, 428, Strand, London, W.C., from the manufacturers, specimens of all new preparations and appliances, which may be brought out from time to time.]

### LIQUOR CARNIS CO. (CAFFYN).

London and Aston Clinton.

We have noticed from time to time the various preparations sent out by the above company, and always with commendation. The company seems to have determined to place before the invalid public a series of food products which has a maximum both of food value and ingenious preparation. These preparations are (1) Liquor Carnis, the pure juice of meat, preserved by a simple carbohydrate, not meat extract, and not a mere solution of albuminous material in salt. The usual tests prove this, and the most important test of all, the use of it for the sick also proves it to be a valuable aid in their treament. (2) Malto Carnis. This is the Liquor Carnis, combined with malt extract and cocoa. It forms a highly nourishing beverage, which has also the power of starch digestion. In actual use, it is an excellent substitute for beef-tea or gruel, or any of the usual fluid nourishing stimulants, and has the very excellent point of not being greasy. (3) Virol. This consists of red and yellow bone marrow, fats, and proteids of raw eggs, egg shells in solution, by means of lemon juice, and extracts of malt. It forms a honey-like emulsion, which contains the food elements in proper proportions in a most digestible form, and, lastly, an exceedingly agreeable flavour. It is an attempt to supply an efficient substitute for cod-liver oil, and, we must add, a successful attempt. As a rule we look on these artificial substitutes with a rather doubtful glance. So often the much-vaunted preparation when tried has turned out a lamentable failure, the patient, and doctor alike deluded with the hopes of at last finding a palatable preparation, and both disgusted when the harshness, saltness, or positively nauseous flavour of the compound has revealed itself. Virol, on the contrary, is a very nice compound, and can be taken by itself or on a biscuit or bread, or even as a cheese cake. We have tried it on several patients with success. A "sans sucre" brand is also made for those who dislike sweet preparations. As will be seen from its formula it is especially adapted for all cases where cod-liver oil used to be given, viz., those with wasting disease, frail children, and over thin babies. 4. Marrol. This consists of substances which make it an analogue of Virol, but it is not so sweet. It contains 17 per cent. of marrow fat, both red and yellow (Virol has in addition the red marrow of the thoracic bones of young calves), combined with malt extract and a phosphate, and flavoured with hop. It will be found an excellent preparation for the same purposes as Virol, but it is not so expensive, and the aromatic flavouring will be better suited to the adult taste. These are, as we noted at the commencement, real advances in invalid feeding. One sees nowadays that the scientific chemist is to a certain extent replacing the domestic cook. Eggs, marrow, powdered malt, hops, have long been known as valuable foods and digestives, but it has been left to the modern chemist's art to so combine them as to get the greatest value out of them. This art exists in no slight measure in taking pains over common materials, and the success of the Liquor Carnis Company is largely due to that homely virtue.

